# Reducing Radiation Dose in Emergency CT Scans While Maintaining Equal Image Quality: Just a Promise or Reality for Severely Injured Patients?

**DOI:** 10.1155/2013/984645

**Published:** 2013-12-05

**Authors:** Ulrich Grupp, Max-Ludwig Schäfer, Henning Meyer, Alexander Lembcke, Alexander Pöllinger, Gero Wieners, Diane Renz, Philipp Schwabe, Florian Streitparth

**Affiliations:** ^1^Department of Radiology, Charité, Humboldt University Medical School, Augustenburger Platz 1, 13353 Berlin, Germany; ^2^Center for Musculoskeletal Surgery, Charité, Humboldt University, Augustenburger Platz 1, 13353 Berlin, Germany

## Abstract

*Objective*. This study aims to assess the impact of adaptive statistical iterative reconstruction (ASIR) on CT imaging quality, diagnostic interpretability, and radiation dose reduction for a proven CT acquisition protocol for total body trauma. *Methods*. 18 patients with multiple trauma (ISS ≥ 16) were examined either with a routine protocol (*n* = 6), 30% (*n* = 6), or 40% (*n* = 6) of iterative reconstruction (IR) modification in the raw data domain of the routine protocol (140 kV, collimation: 40, noise index: 15). Study groups were matched by scan range and maximal abdominal diameter. Image noise was quantitatively measured. Image contrast, image noise, and overall interpretability were evaluated by two experienced and blinded readers. The amount of radiation dose reductions was evaluated. *Results*. No statistically significant differences between routine and IR protocols regarding image noise, contrast, and interpretability were present. Mean effective dose for the routine protocol was *25.3* ± 2.9 mSv, *19.7* ± 5.8 mSv for the IR 30, and *17.5* ± 4.2 mSv for the IR 40 protocol, that is, 22.1% effective dose reduction for IR 30 (*P* = 0.093) and 30.8% effective dose reduction for IR 40 (*P* = 0.0203). *Conclusions*. IR does not reduce study interpretability in total body trauma protocols while providing a significant reduction in effective radiation dose.

## 1. Introduction

The use of computed tomography (CT) brought enormous benefits to modern medicine and diagnostic CT examinations are increasingly used in recent years because of their speed, availability, and diagnostical power. In particular, for patients with polytrauma during the early resuscitation phase, whole-body CT is recommended as the standard diagnostic modality [1]. However, the common utilization of CT is accompanied by a steady increase in the population's cumulative exposure to ionizing radiation [2, 3]. As X-rays have been classified as “carcinogen,” new efforts to minimize radiation exposure were undertaken to meet rising concerns of possible long-term cancer, especially regarding pediatric and young patients as well as patients undergoing several follow-up CT examinations [4]. A plurality of approaches, from “AEC” (automated exposure control) to “X-ray beam collimation,” led to a significant reduction in radiation dose [5, 6]. With the fast advancement of computational power, the technique of iterative reconstruction (IR), well known from SPECT and PET imaging, became the center of attention for CT adaption in recent years [7–10].

The group of severely injured patients is of great concern for dose reduction as these patients may be of a young age and standard protocols for emergency settings use relatively high radiation doses in order to detect subtle but possibly life-threatening lesions [11]. As recently demonstrated, IR algorithms do not significantly delay CT imaging time in an emergency setting [12], though the impact of IR on image quality and dose reduction has not been investigated.

The working hypothesis was that the use of IR algorithms would reduce effective radiation doses without affecting image quality and interpretability in comparison with routine CT imaging based on filtered back projection (FBP). Therefore this study aimed to prospectively evaluate different levels of IR algorithms on imaging quality and dose reduction for a proven CT full body trauma protocol.

## 2. Materials and Methods

### 2.1. Study Design

The study was carried out prospectively. The study design was approved by the institutional ethics board. The need for informed consent was waived as patients were not exposed to an additional radiation dose and patient data was anonymized. The study was conducted at a university teaching hospital.

All examined patients were classified as severely injured patients (injury severity score (ISS) ≥16) by the emergency department and underwent CT scans within the scope of the routine in-house algorithm for patients with severe and multiple injuries in accordance with the currently valid guideline [13]. At the time of study analysis, 10 patients were examined with IR 30 and 16 patients with IR 40 protocol. For matching the patients in all three patient groups (FBP, IR 30, and IR 40) these patients were compared by scan range and maximal abdominal diameter. Subsequently, some patients had to be excluded and 6 patients per group were enrolled in the final statistical analysis (age = 53.9 ± 19.9 years, 5 females). All groups were matched manually as to scan range and maximal abdominal cross-section area to attain a homogenous study collective. The authors regarded this approach to be a more accurate approximation for full body radiation exposure of the study group than traditional parameters, that is, BMI [14]. The control group was examined with the routine filtered back projection (FBP) protocol whereas the protocol for the first study group, performed on the same CT scanner, consisted in 30% adaptive statistical iterative reconstruction (ASIR) application in the raw data material. After a preliminary assessment of the newly acquired image quality and positive results in terms of interpretability, a stronger level of IR (40) was implemented on the standard protocol and performed on the second study group.

### 2.2. CT Protocol

All patients were examined using a 64-slice multidetector CT scanner (Lightspeed VCT, GE Healthcare, USA). The emergency protocol consists of two separate scans. The first is an axial scan of the cranium, angulated between 0° and 30° (depending on the positioning of the patient) without injection of a contrast agent (CA) in order to detect possible intracranial bleeding.

Second, after application of CA (see Section 2.3) a helical scan of the whole body is performed in craniocaudal direction, ranging from above the frontal sinus (in order to picture the cerebral arterial circle) to the bottom of the pelvis. Image acquisition is conducted with the following parameters: tube voltage: 140 kV, collimation: 40, pitch: 1,375, and noise index: 15.

The protocol of the axial cranium scan was not modified. If additional scans were performed due to individual injury patterns, these have not been considered and were excluded from the image quality and radiation dose analyses.

### 2.3. Injection Table

For the helical whole-body spiral 140 mL of CA was administered in a split bolus technique:

 100 mL CA (2 mL/s flow rate),
 20 mL saline (1 mL/s flow rate),
 60 mL CA (4 mL/s flow rate),
 40 mL saline (4 mL/s flow rate).

The CT scan starts with a delay of 85 seconds after first injection of the 100 mL CA.

Using this technique, an additional scan and radiation dose can be avoided as arterial and venous contrasting is depicted in the same scan. The nonionic, low osmolality contrast medium iobitridol (Xenetix 350, Guerbet GmbH, Germany) was utilized as CA.

### 2.4. Data Reconstruction

IR algorithms attempt to overcome elevated image noise and artifacts resulting from reduced voltage and current. The adaptive statistical iterative reconstruction (ASIR) technique attempts to accurately rebuild images by concentrating on noise reduction [15]. It therefore uses information obtained from the FBP algorithm as an initial building block for image reconstruction. The ASIR model then uses matrix algebra to transform the measured value of each pixel (*y*) to a new estimate of the pixel value (*y*′). This pixel value is then compared with the ideal value that the noise model predicts. This process is repeated in successive iterative steps until the final estimated (*X*) and ideal pixel values ultimately converge [16, 17]. Using this method, IR algorithms are able to selectively identify and then subtract noise from an image.

The image acquisition was modified by 30% (40%) use of IR in the raw data domain. Due to computational limitations the raw data material is then first computed into slices of 5 mm thickness in order to deliver a fast summary of the patient for diagnostical assessment and subsequently into slices of 0,625 mm thickness. The image reconstruction of the 5 mm slices used the same level of ASIR algorithms (30/40) as those of the raw data whereas the thin slices were both computed with 60% ASIR to further reduce image noise. No change of radiation dose is linked to the reconstruction ASIR.

### 2.5. Data Analysis

Quantitative analysis of image quality was evaluated for noise, that is, the standard deviation (SD) of attenuation value. Therefore a region of interest (ROI) was drawn as large as possible in the supracarinal trachea without exceeding the lumen. Qualitative analysis of the acquired images was performed by two experienced and blinded radiologists in consensus. The images were cleared of all technical information in order to reduce expectation bias.

Image quality was evaluated in five categories: noise, contrast, artifacts, detectability of small structures, and overall diagnosability. Each category was evaluated by using a five-point Likert scale with 5 representing the best possible result and 1 insufficient results, for example, for overall diagnosability: (1) nondiagnostic image quality, (2) severe blurring with uncertainty about the evaluation, (3) moderate blurring with restricted assessment, (4) slight blurring with unrestricted diagnostic image evaluation possible, and (5) excellent image quality, no artifacts.

### 2.6. Statistical Analysis

The data were analyzed using SPSS 18.0 (SPSS Inc., Chicago, Ill). Radiation doses and image quality parameters were compared using the Mann-Whitney-*U* test. A *P* value of less than 0.05 was considered a statistical significance.

## 3. Results

### 3.1. Image Quality

Quantitative analysis for image noise (region of interest in the supracarinal trachea) yielded the following results: the standard deviations of attenuation value measured in HU were 3.9 ± 0.7, 3.4 ± 0.8, and 3.3 ± 0.6 for the routine, IR 30, and IR 40 protocol. There were no significant differences between the three groups.

**Figure 1:**
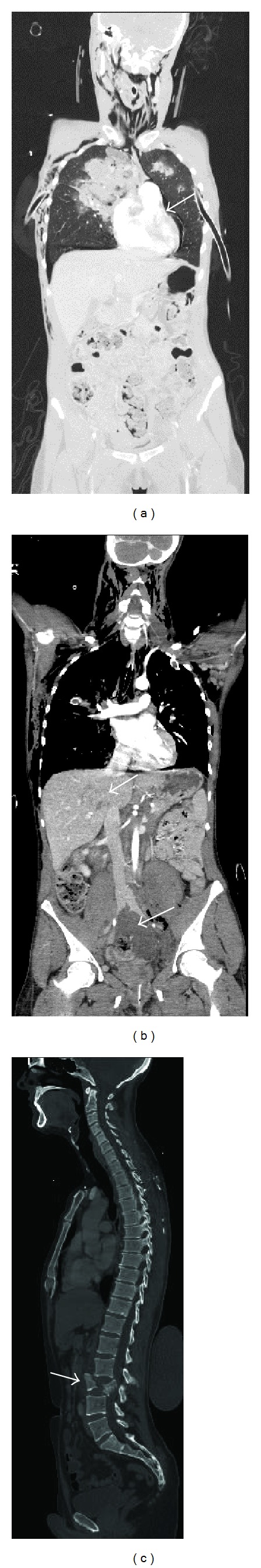
25-year-old female patient with a heavy trauma falling from a roof with suicidal intention. Image acquisition with the CT ASIR 40 protocol. (a): coronal orientation/lung window showing bilateral pneumothoraces (arrow) with Bülau drains in situ, heavy contusion in both lungs, and extensive thoracal and cervical soft tissue emphysema. (b): coronal orientation/soft tissue window showing hypodensities in terms of an acute liver haematoma in the right lobe (arrow). Also note a huge retroperitoneal haematoma in the lower abdomen (arrow). (c): another male 42-year-old patient after falling from a roof. CT ASIR 40 protocol, sagittal plane/bone window. Vertebral body fracture in L3 with consecutive spinal stenosis (arrow).

**Figure 2:**
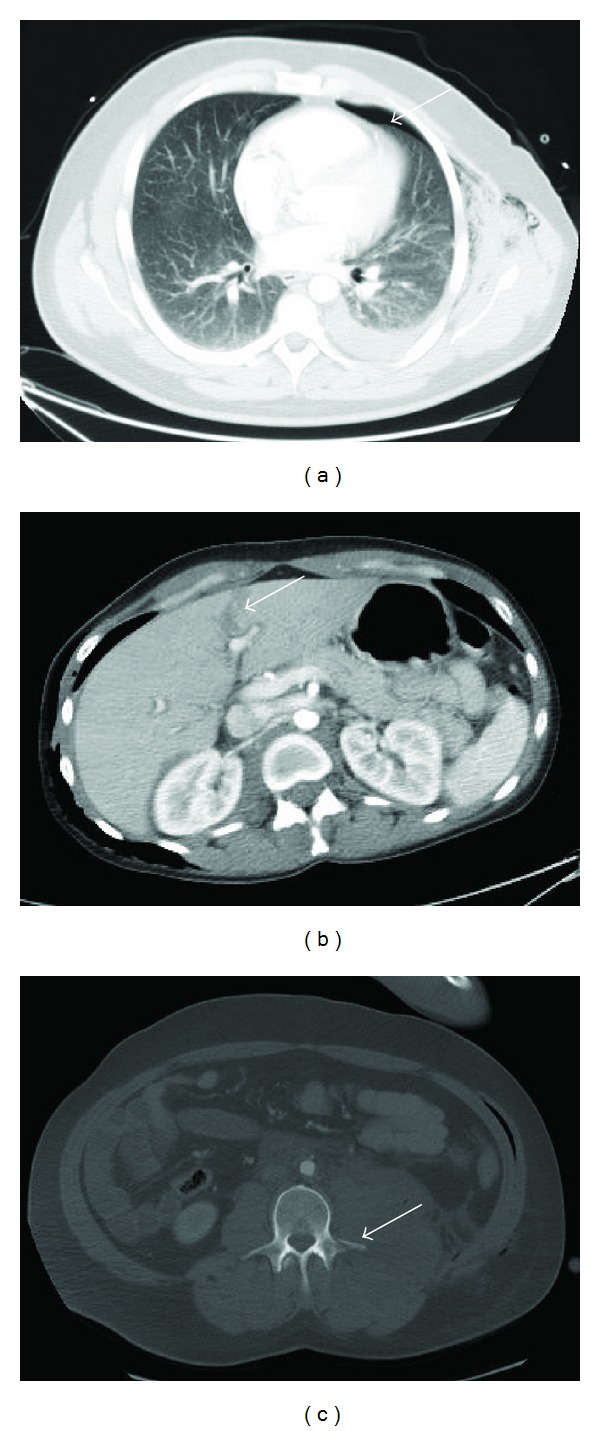
Image acquisition with the routine CT protocol reconstructed by filtered back projection (FBP) without IR. In (a) the white arrow points to a small pneumothorax in the lingula segment. In (b) a hepatic laceration in liver segments 2 and 4a is displayed. Notice the semicircular soft tissue emphysema on the right side. In (c) the arrow points to the fracture of the left transverse process of L3 with no major dislocation and a corresponding haematoma of the psoas muscle.

**Figure 3:**
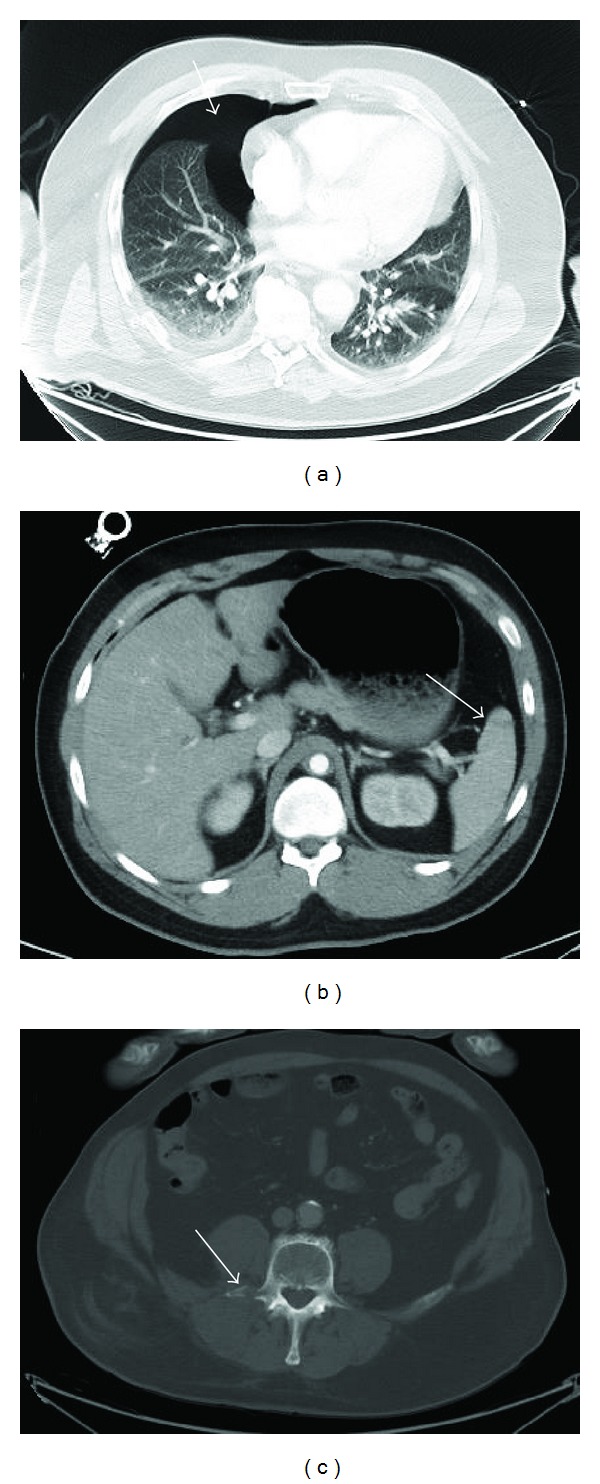
Image acquisition with the IR 30 protocol. In (a) the arrow points to a major ventral pneumothorax of the right lung. In the dorsal area there is pleural effusion with adjacent dystelectasis. In (b) a subtle laceration of the ventral splenic pole is marked by the arrow. Similar to the FBP protocol in Figure 1, a fracture of a transverse process of L4 without major dislocation can be diagnosed on the right side.

**Figure 4:**
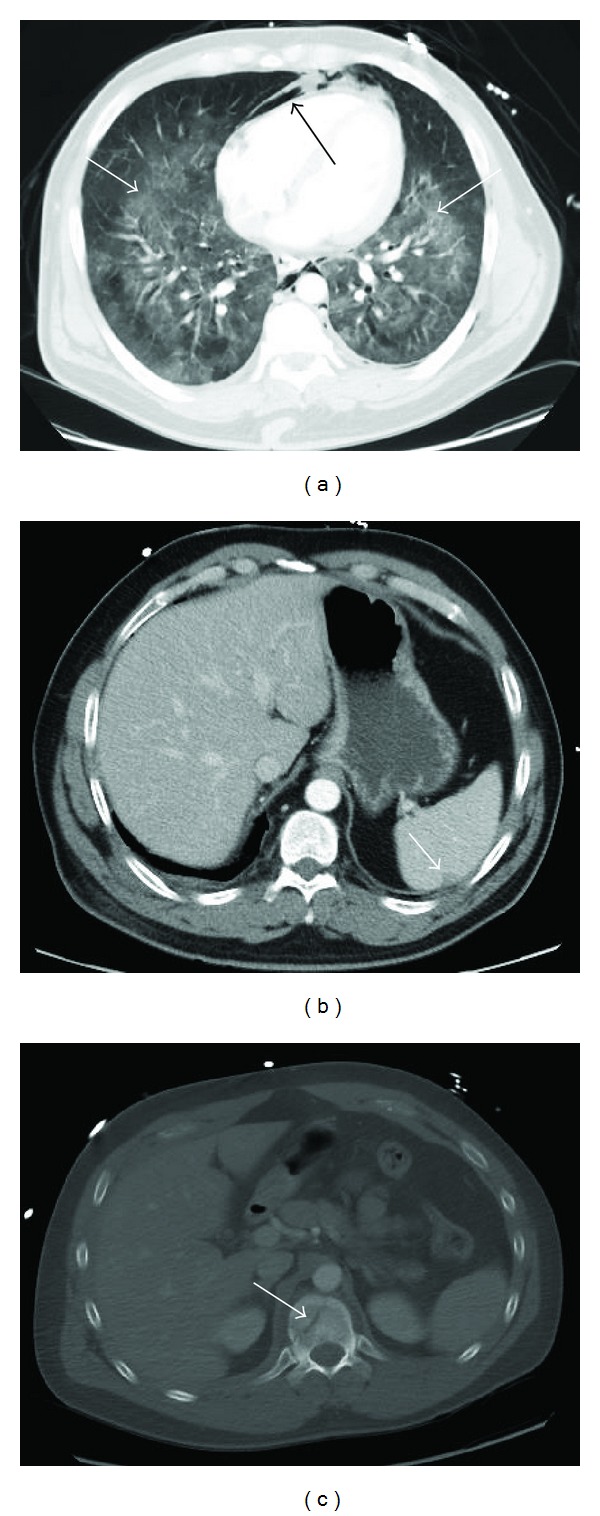
Image acquisition with the IR 40 protocol. In (a) the white arrows mark the bipulmonary contusions. The black arrow tags the mediastinal emphysema. In (b) the white arrow marks a subtle splenic laceration in the dorsal pole similar to the splenic lesion in Figure 3(b). Finding of a vertebral body fracture in (c) after a motorcycle accident. No accompanying lesion of the pancreas could be found.

Qualitative analysis yielded the following results: all (*n* = 18) CT examinations were estimated to be of excellent image quality without artifacts compromising diagnosability (level 5) in terms of image artifacts, detectability of small structures, and overall diagnosability (Figures 1, 2, 3, and 4). Regarding image noise and image contrast, no statistically significant differences resulted, with image noise estimated at 4.7 ± 0.5, 5 ± 0, and 4.8 ± 0.4 for the FBP, IR 30, and IR 40 protocol. For image contrast the results were 5 ± 0, 4.8 ± 0.4, and 4.8 ± 0.4, respectively. There were no significant differences between the three groups.

### 3.2. Radiation Dose

**Figure 5:**
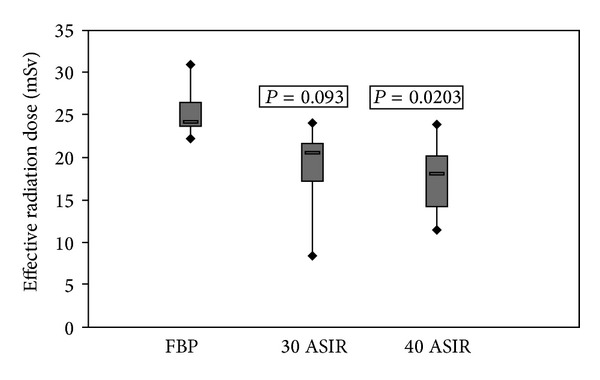
Dose reduction, comparison of three different CT protocols. The mean effective dose for the standard (FBP) protocol was 25.3 ± 2.9 mSv, for IR 30 19.7 ± 5.8 mSv, and 17.5 ± 4.2 mSv for IR 40 protocol, that is, 22.1% effective dose reduction for IR 30 (*P* = 0.093) and 30.8% effective dose reduction for ASIR 40 (*P* = 0.0203). A *P* value of less than 0.05 was considered a statistical significance.

To estimate the effective radiation dose, first the CT volume dose index (CTDIvol) and the dose-length product (DLP) were obtained from the electronically stored dose report of each performed CT scan. The effective radiation dose was then calculated by multiplying the DLP by a conversion coefficient *k* of 0.017 mSv/mGy·cm [18]. See Table 1 for an overview of the radiation dose. The mean effective dose for the standard FBP protocol was 25.3 ± 2.9 mSv; the implementation of IR 30/30 resulted in 19.7 ± 5.8 mSv and in 17.5 ± 4.2 mSv for IR 40/40 protocol, that is, 22.1% effective dose reduction for IR 30 (*P* = 0.093) and 30.8% effective dose reduction for IR 40 (*P* = 0.0203). See Figure 5 for an overview of the dose reduction.

## 4. Discussion

After its introduction in 1973, computed tomography (CT) was fast accepted by the medical community. In particular in the treatment of severely injured patients its diagnostic accuracy brought immense benefits and whole-body CT scans have become an integral part of the Advanced Trauma Life Support (ATLS) in multiple trauma centers. As several multicenter studies showed, whole-body CT scans in the early phase of treatment resulted in a significant increase of survival [1, 18, 19]. Hence, benefits of CT in an emergency setting outweigh by far the downsides with exposure to ionizing radiation being the most critical disadvantage. With a median cumulative effective dose as high as 40.2 mSv for CT scans of blunt trauma patients [11], efforts to reduce effective radiation doses still constitute a primary objective. Since the first introduction of IR algorithms into CT imaging there have been numerous studies regarding image quality, noise, and radiation dose reduction [20–22]. The overall findings, depending on the study design, proved either a significant dose reduction, a better image quality, or both. However, the applicability of these algorithms to an emergency setting has not yet been examined, as image quality is of the highest priority in these circumstances.

With our findings being a first evaluation of the impact of IR algorithms in an emergency setting, a number of issues have to be addressed in the future.

It has been shown in the literature that the mean effective dose for full body CT examinations ranges between 14 and 21 mGy whereas the mean effective dose in the control group of our study was 25 mGy [23]. The effective radiation dose is slightly above average as we used 140 kV for the routine trauma protocol. This is based on the fact that as a national center of maximum care we are confronted by a tendency to receive more complex severely injured patients than other institutions and image quality is paramount. After careful considerations of advantages and disadvantages, our institution decided to apply 140 kV for the routine whole-body trauma protocol, as this protocol is more robust concerning artifacts from foreign bodies (e.g., equipment from anesthetic intensive care). Of course, the use of a protocol with 120 kV is also appropriate. It can be assumed that the implementation of 120 kV for whole-body protocols instead of 140 kV will lead to a further reduction in radiation dose. The impact of IR on a 120 kV protocol has to be addressed in future studies.

As mentioned earlier, we first implemented an IR level of 30% in the routine protocol to ensure an excellent image quality and only afterwards modified the protocol to ASIR 40. As our study demonstrates there is no compromise to the diagnosability and it seems justifiable to investigate the implementation of ASIR 50 (representing the highest level of ASIR) to whole-body protocols in an emergency setting.

As stated above, the full body trauma protocol consists of two scans, an axial cranium scan and a helical body spiral. In this study only the latter was modified with IR algorithms as to our knowledge the impact of IR algorithms on the image quality for the neurocranium has not yet been evaluated outside an emergency setting. Nonetheless, mean DLP for routine protocols was 2273.0 mGy·cm, whereas mean DLP for the helical spiral was 1491.4 mGy·cm, thus accounting only for about 66% of total radiation dose; that is, axial cranium scans account for about one-third of radiation dose. Whether or not IR algorithms are applicable for cranium scans has to be investigated by future studies.

### 4.1. Limitations

With this study designed to be a feasibility evaluation of IR algorithms in an emergency setting, the major limitation naturally consists in the small number of patients examined. Secondly, there is no possibility of intraindividual comparison of image quality and radiation dose. This obstacle could be limited with a manually selected study group with respect to scan range and abdominal cross-section diameter. Nonetheless, a series of validity impairments, that is, limitations in the positioning of patients, foreign bodies in the FOV, et cetera, may have resulted in increased radiation doses due to beam-hardening artifacts.

Thirdly, the small study group did not allow for evaluation of similar injury patterns. Fourthly, as the sole parameter for objective image quality, we only assessed the image noise. Further studies have to be performed in a larger patient collective investigating different contrast-to-noise ratios.

## 5. Conclusions 

In these preliminary results, the use of IR algorithms is a promising application to reduce radiation exposure without compromise to the radiological interpretability, even in an emergency setting where image quality is paramount.

## Figures and Tables

**Table 1:** Radiation dose.

Parameters	Standard 140 kV	IR 30 140 kV	IR 40 140 kV
Scan range (cm)	90.1 ± 3.2	90.9 ± 4.9	90.8 ± 3.4
Max. abdominal cross-section			
Coronal (cm)	35.2 ± 3.7	37.2 ± 3.1	36.3 ± 3.3
Sagittal (cm)	27.5 ± 3.3	27.2 ± 4.7	26.8 ± 4.4
CDTI_vol_ (mGy·cm)	15.5 ± 1.8	12.1 ± 3.5	10.7 ± 2.5
DLP (mGy/cm)	1491.4 ± 160.1	1179.1 ± 354.6	1035.4 ± 229.1
Effective radiation dose (mSv)	**25.3 ± 2.9**	**19.7 ± 5.8**	**17.5 ± 4.2**

Parameters of the routine FBP protocol, IR 30 protocol, and IR 40 protocol.

Data are presented as mean ± standard deviation. CTDIvol: CT volume dose index, DLP: dose-length product.
